# Clinical Risk Index for Babies Scoring System in Prediction of Mortality in Premature Babies: A Prospective Study at in Mumbai

**DOI:** 10.4314/ejhs.v36i1.9

**Published:** 2026-01

**Authors:** Nilima Narkhede, Rajaram Weling, Reshma Pradhan

**Affiliations:** 1 Department of Pediatrics, Bharatratna Dr Babasaheb Ambedkar Municipal General Hospital, Kandivali(w), Mumbai

**Keywords:** CRIB II score, neonatal mortality, low birth weight, preterm infants, NICU

## Abstract

**Background:**

The Clinical Risk Index for Babies II (CRIB II) is a validated scoring system for predicting early mortality risk among low birth weight (LBW) preterm neonates. However, its routine use remains limited in many developing countries. This study aimed to evaluate the effectiveness of the CRIB II score in predicting neonatal mortality among LBW infants admitted to a tertiary care Neonatal Intensive Care Unit (NICU).

**Methods:**

This prospective cohort study included 140 preterm neonates (gestational age 23–32 weeks) admitted to the NICU within 24 hours of birth. Baseline demographic variables, clinical characteristics, and CRIB II scores were recorded Neonatal outcomes were followed until discharge or death. Diagnostic performance was assessed using sensitivity, specificity, predictive values, accuracy, and receiver operating characteristic (ROC) curve analysis.

**Results:**

There was a slight male predominance (51%). The mean birth weight was 1550.97 ± 253.58 g, and the mean gestational age was 30.42 ± 2.23 weeks. Overall neonatal mortality was 32.85%. Mortality increased progressively with higher CRIB II scores: 0% in level I, 3.84% in level II, 64.70% in level III, and 100% in level IV. The CRIB II score demonstrated excellent predictive performance with a sensitivity of 84.78%, specificity of 90.42%, and diagnostic accuracy of 88.57%.

**Conclusion:**

The CRIB II score is a reliable and effective tool for early prediction of neonatal mortality in LBW preterm infants and should be considered for routine risk stratification in NICU settings.

## Introduction

Neonatal mortality remains disproportionately high in low- and middle-income countries, with prematurity, low birth weight, and maternal risk factors such as diabetes, infections, and hypertensive disorders being major contributors. Additional factors including hypoxic-ischemic injury, infections, and mode of delivery further influence neonatal outcomes (1).

Accurate assessment of illness severity in the NICU is essential for predicting mortality risk, optimizing resource allocation, and improving quality of care. Standardized scoring systems allow objective quantification of neonatal condition and facilitate comparison across populations and care settings (2,3). Among these, CRIB II is specifically designed for infants born at <32 weeks of gestation and incorporates birth weight, gestational age, admission temperature, base excess, and sex (4–6).

## Materials and Methods

**Study design and population**: This prospective cohort study was conducted over 10 months (November 2022 to August 2023) in the Department of Paediatrics at a Level III NICU in Mumbai. Ethical approval was obtained, and written informed consent was secured from parents or guardians.

A total of 140 preterm neonates with gestational age between 23 and 32 weeks, admitted within 24 hours of birth, were included. Neonates with birth weight <500 g, major congenital anomalies, genetic disorders, surgical emergencies, delivery-room deaths, late admissions (>12 hours), or lack of parental consent were excluded.

**CRIB II scoring**: CRIB II scores (range 0–27) were calculated by a single observer using birth weight, gestational age, admission temperature, base excess, and sex (4-6).

Neonates were categorized as follows:

- **Level I**: CRIB II score 1–5
- **Level II**: CRIB II score 6–10
- **Level III**: CRIB II score 11–15
- **Level IV**: CRIB II score > 15

**Sample size calculation**: In a previous prospective cohort study 140 neonates were included who were admitted during their first 24 hours of birth.

**Statistical analysis**: Data were analysed using SPSS version 23. Continuous variables were expressed as mean ± SD, and categorical variables as frequencies and percentages. Diagnostic performance was assessed using sensitivity, specificity, predictive values, accuracy and ROC curve analysis. A p-value <0.05 was considered statistically significant.

## Results

Of the 140 neonates, as seen in Table 1, 72 (51%) were male and 68 (49%) were female. The mean birth weight was 1550.97 ± 253.58 g, and the mean gestational age was 30.42 ± 2.23 weeks. The most common comorbidities were intracranial hemorrhage and hypoxic-ischemic encephalopathy (8.57% each). Lower segment caesarean section was the most frequent mode of delivery (65%).

**Table 1:** Baseline characteristics of preterm neonates admitted to the NICU (n = 140)

Variable	Frequency (%)/Mean ± SD
Male sex	72 (51.0)
Birth weight (g)	1550.97 ±253.58
Gestational age (weeks)	30.42 ±2.23
Birth weight 1501–2000 g	66 (47.14)
Intracranial hemorrhage	12 (8.57)
Hypoxic-ischemic encephalopathy	12 (8.57)
LSCS delivery	91 (65.0)

The mean CRIB II score was 10.1 ± 3.9 (range 1–19). Overall neonatal mortality was 32.85%. Mortality increased significantly with increasing CRIB II score category.

The CRIB II score demonstrated a sensitivity of 84.78%, specificity of 90.42%, positive predictive value of 81.25%, negative predictive value of 92.39%, and an overall diagnostic accuracy of 88.57%.

ROC curve analysis identified a CRIB II score ≥11 as the optimal cut-off for predicting neonatal mortality.

**Figure 1:**
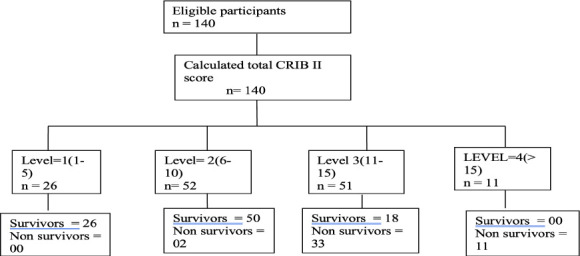
Distribution of neonates according to CRIB II score categories and corresponding mortality rates

Figure 1. Receiver operating characteristic (ROC) curve showing diagnostic performance of CRIB II score for prediction of neonatal mortality.

## Discussion

In the present study, 140 preterm neonates were analysed, with a slight male predominance (51% male and 49% female). The mean birth weight was 1550.97 ± 253.58 g, and the mean gestational age was 30.42 ± 2.23 weeks. These findings are comparable to those reported by Saied DA et al^7^ and Rastogi PK et al^8^. Preterm birth remains the leading direct cause of neonatal mortality worldwide, accounting for approximately 35% of the estimated 3.1 million neonatal deaths annually, and is the second most common cause of death among children under five years of age after pneumonia. Male preterm infants have been reported to outnumber females and to exhibit greater susceptibility to mortality (6).

Most neonates had an admission temperature between 31 °C and 34 °C, with a mean temperature of 34.6 ±1.4 °C. The mean base excess was −11.5 ± 6.0 mmol/L, reflecting severe metabolic acidosis, a finding consistent with previous studies. The most frequently observed comorbidities were intracranial hemorrhage and hypoxic-ischemic encephalopathy (HIE), each affecting 8.57% of neonates. In addition, 65% of neonates were delivered by lower segment caesarean section (LSCS). Maternal infections were documented in 12.85% of cases, while 10% of mothers had preeclampsia or eclampsia, findings that are consistent with those reported by Stomnaroska O et al^1^ and Marete IK et al^9^.

Neonates were stratified using the CRIB II score, with a mean score of 10.1 ± 3.9. This distribution aligns with findings from earlier studies and further supports the usefulness of the CRIB II score in predicting neonatal outcomes (6,7,9–12). As showed in table 2, marked increase in mortality was observed with rising CRIB II score categories, with mortality rates of 0% in level I, 3.84% in level II, 64.70% in level III, and 100% in level IV. These findings indicate that neonates with higher CRIB II scores are at substantially increased risk of mortality and therefore require intensified clinical monitoring and intervention.

**Table 2:** Neonatal mortality according to CRIB II score category

CRIB II level	Score range	Mortality (%)
Level I	1–5	0
Level II	6–10	3.84
Level III	11–15	64.70
Level IV	>15	100

The study further demonstrated that lower gestational age, lower birth weight, hypothermia at admission, shorter duration of hospital stay, and more severe metabolic acidosis were associated with increased mortality. These observations are comparable to findings reported by Ezz-Eldin ZM et al^6^ and Gagliardi L et al^13^. The predictive performance of the CRIB II score was evaluated using receiver operating characteristic (ROC) curve analysis. As showed in table 3,The CRIB II score showed superior diagnostic performance compared with birth weight and gestational age alone, with a sensitivity of 84.78%, specificity of 90.42%, and an overall diagnostic accuracy of 88.57%. These results highlight the CRIB II score as a more robust predictor of neonatal mortality than traditional standalone variables.

**Table 3:** Diagnostic performance of CRIB II score in predicting neonatal mortality

Parameter	Value (%)
Sensitivity	84.78
Specificity	90.42
Positive predictive value	81.25
Negative predictive value	92.39
Diagnostic accuracy	88.57

Moreover, the sensitivity, specificity, and predictive values of the CRIB II score were higher than those of traditional models when used independently. The area under the ROC curve for predicting mortality was also greater for the CRIB II score than for birth weight or gestational age alone, findings that are consistent with previous studies (6,9,10,12–15). Despite these strengths, the study has limitations, including its single-centre design and relatively small sample size, which may limit the generalizability of the findings. Nonetheless, the results underscore the potential value of the CRIB II score in guiding clinical decision-making in neonatal intensive care units.

In conclusion, the CRIB II score is a valuable and reliable tool for predicting mortality risk among low-birth-weight preterm neonates. Its strong diagnostic performance facilitates early identification of high-risk infants and supports timely clinical intervention. Given its superior predictive accuracy, the CRIB II score should be preferred over traditional models for assessing neonatal outcomes in NICU settings.

## References

[1] Stomnaroska O, Danilovski D (2020). The CRIB II (Clinical risk index for babies II) score in prediction of neonatal mortality. CONTRIBUTIONS. Sec. of Med. Sci.

[2] Dorling IS, Field DI, Manktelow B (2005). Neonatal disease severity scoring systems Archives of Disease in Childhood. Fetal and Neonatal Edition.

[3] Masoumehmohkam, Afjeii Abolfazl, Payandeh Paiam (2011). A comparison of crib ii, snap, snapii and snap-pe score for prediction of mortality I critically ill neonates. Medical journal of the Islamic republic of iran.

[4] Kadivar M, Sagheb S, Bavafa F (2007). Neonatal Mortality Risk Assessment in a Neo). Iranintensive Care Unit (NICU). Iran J Pediatr.

[5] De Felice C, Vecchio AD, Latini G (2005). Evaluating illness severity for very low birth weight infants: CRIB or CRIB-II?. The Journal of Maternal-Fetal and Neonatal Medicine.

[6] Ezz-Eldin ZM, Hamid TA, Youssef MR, Nabil Hel-D (2015). Clinical Risk Index for Babies (CRIB II) Scoring System in Prediction of Mortality in Premature Babies. J Clin Diagn Res.

[7] Saied DA (2005). Evaluation of Neonatal Mortality Risk Predictors Among Premature Neonates. Alexandria Journal of Pediatrics.

[8] Rastogi PK, Sreenivas V, Kumar N (2010). Validation of CRIB II for prediction of mortality in premature babies. Indian Pediatr.

[9] Marete I, Wasunna A, Otieno P (2011). Clinical risk index for babies (CRIB) II score as a predictor of neonatal mortality among low-birth-weight babies at Kenyatta National Hospital. East Afr Med J.

[10] Rehman A, Hamid MH (2022). Accuracy of CRIB II score in predicting the neonatal mortality in very preterm babies. P J M H S.

[11] Mishal M, Noor N, Khan S (2019). Crib (Clinical Risk Index for Babies) Scoring System in Prediction of Mortality in Premature Babies. Indo Am. J. P. Sci.

[12] Qasim S, Zahid S, Islam A, Anwar M, Siddique S, Rafique A (2022). Clinical Risk Index Score (CRIB II) as a Predictor of Neonatal Mortality among Premature Babies. PJMHS.

[13] Gagliardi L, Cavazza A, Brunelli A, Battaglioli M (2004). Assessing mortality risk in very low birth weight infants: a comparison of CRIB; CRIBII and SNAPPE-II. Arc Dis Child Fetal Neon Ed.

[14] Akbar N, Sarwar S, Sajjad M, Hayat M, Hassan KA, Wasim A (2020). The Accuracy of CRIB II for Predicting Mortality of Severely ill Preterm Neonates. APMC.

[15] Jašić Mladen, Dessardo Nada Sindičić, Dessardo Sandro, Rukavina Koraljka Manestar (2016). CRIB II score versus gestational age and birth weight in preterm infant mortality prediction: who will win the bet. Signa Vitae.

